# Nano-Pulse Stimulation Therapy Initiates Regulated Cell Death in Skin, Unlike Bovie Radiofrequency Ablation and Cryoablation

**DOI:** 10.1089/bioe.2024.0008

**Published:** 2024-05-23

**Authors:** Richard Nuccitelli, Michelle Martinez, David Kaufman, Darius Mehregan, Lauren Johnston, William A. Knape

**Affiliations:** ^1^ Pulse Biosciences, Inc., Hayward, California, USA.; ^2^ Kaufman and Davis Plastic Surgery, Folsom, California, USA.; ^3^ Department of Dermatology, Wayne State University, Detroit, Michigan, USA.

**Keywords:** nano-pulse stimulation, nanosecond, nanosecond pulsed electric fields, regulated cell death, cryoablation, Bovie^®^ radiofrequency ablation

## Abstract

**Background:**

This study describes a unique new bioelectric approach for clearing skin lesions and illustrates the clinical and histological differences between this new method and the standards of cryoablation and Bovie^®^ radiofrequency ablation (RFA).

**Objectives:**

To determine the advantage of stimulating regulated cell death with nanosecond pulsed electric fields over the necrosis response elicited by thermal ablation modalities.

**Methods:**

Human abdominal skin was treated with cryoablation, Bovie^®^ RFA, and nano-pulse stimulation (NPS) therapy four times before an abdominoplasty procedure was performed to collect skin for histology. The clinical appearance and corresponding histology of each treatment were documented over time and compared.

**Results:**

NPS therapy triggered regulated cell death as indicated by the appearance of activated Caspase-3 at 2 h post treatment and the absence of nuclear staining 1 day post treatment. Epidermal regeneration follows without impacting the noncellular dermis in contrast to cryoablation and Bovie^®^ RFA which trigger necrosis and often cause scarring, inflammation, or permanent pigmentary changes. The main differences between NPS therapy and other ablation modalities are the level of fibrosis, amount of scarring, elastic fiber concentration, and inflammation. An analysis of the skin thickness 30 days after the treatment indicates that NPS-treated skin is the most similar to untreated skin but cryoablated and RF-ablated skin were 2- and 3.5-fold thicker, respectively, suggesting that they initiate necrosis rather than regulated cell death.

**Conclusions:**

We conclude that NPS therapy is a unique nonthermal modality that may be applied for clearing benign skin lesions by initiating the skin’s own programmed cell death pathway instead of necrosis as used by cryoablation and Bovie^®^ RFA.

## Introduction

The bioelectric properties of the largest organ in our body, the skin, are not widely known. These properties are directly involved in the ability of our skin to heal wounds, one of the most important characteristics of this organ. Skin generates a transepithelial potential of 20–60 mV across itself, inside positive, and this drives current flow along the inner surface of the epidermis and out of any wounds in the skin, generating a subepidermal electric field that points toward the wound.^1^ This electric signal guides keratinocytes and white blood cells to the wound and plays a critical role in wound healing.^2^ In this study, we describe a nonthermal electric signal that triggers regulated cell death in abdominal skin and can be used to ablate skin lesions. We also compare this skin response with that resulting from two other ablation modalities, radiofrequency ablation (RFA) and cryoablation, to clarify the difference between the skin responses.

The removal of skin lesions has undergone several technical advances over the past 50 years. Most of these involved the introduction of thermal ablation tools, including Bovie^®^ RFA, cryoablation, and lasers. Thermal ablation applies extreme temperatures inside of target tissues to levels that denature proteins and triggers necrosis.^3,4^ Two drawbacks to this approach are the spread of heat from the application point to damage surrounding tissues, as well as the permanent damage to vessels and nerves in the target region. However, a new modality called Nano-Pulse Stimulation™ (NPS™) Therapy uses ultra-short pulses of electric energy to initiate regulated cell death in target cells in a highly localized manner.^5,6^ This new nonthermal therapy is explained in detail here with a direct comparison of its effect on abdominal skin with those of cryoablation and Bovie^®^ RFA.

## Mechanism Used by NPS Therapy

### Electroporation

Pulsed electric fields have been used for decades to permeabilize cells. Permeabilization requires the formation of pores through the lipid bilayer that will allow molecules in the aqueous solution to cross that barrier. Lipid bilayers generally exhibit low water permeability making them effective barriers in an aqueous environment. However, because water exists in solution as a dipole with a net positive charge, it will move in response to an imposed electric field. With electric field application, the charged water molecules near the outer plasma membrane of cells will be pushed up against the membrane but will not penetrate the membrane until the field reaches the “breakdown potential” of about 400–500 mV/membrane. This was referred to as “electroporation” by Neumann.^7^ Molecular dynamics models of cellular membranes have demonstrated that pulsed fields of this magnitude push water molecules sufficiently far into the lipid bilayer to form a water-filled pore or defect in the membrane. This defect will allow charged molecules to move across the bilayer which is the definition of permeabilization. Generally speaking, the longer the electric pulse, the larger the pore size that is generated in the membrane. The earliest pulsed fields used were in the microsecond and millisecond domain and generated pores large enough to allow the transport of at least 40 kDa molecules across the plasma membrane.^8^ In contrast, NPS therapy uses pulses that are 1000-fold shorter in the nanosecond range and these generate transient nanopores only about 1 nm wide and limit transport to much smaller molecules less than 1 kDa, such as ions.^9^ However, because much cellular signaling involves ion transport, permeabilization provides a mechanism by which NPS therapy can influence cell function.

### Nanopore formation in both the plasma membrane and organelle membranes

The evidence for the formation of nanopores initially came from the measurement of a spike in intracellular Ca^2+^ concentration with each nanosecond pulse^10^ followed by patch clamp data measuring the current flow through the nanopores^11^ and the determination of molecular sizes capable of traversing the nanopores.^12^ These studies indicated that pulses in the nanosecond domain could generate transient, 1 nm wide nanopores in the plasma membrane and endoplasmic reticulum membranes through which ions could move down their normal concentration gradient. Because Ca^2+^ spikes are involved in many aspects of cellular signaling such as neuromuscular transmission and cell division, the ability of NPS to initiate these spikes provides a unique means to influence cellular functions.

In addition to permeabilizing the plasma membrane, these early investigations found that NPS treatment could permeabilize intracellular organelles such as vesicles,^13^ the endoplasmic reticulum,^14^ and mitochondria.^15^ A recent electron microscopy study also confirmed this organelle permeabilization.^16^ This ability to permeabilize small intracellular organelles was new and is a unique characteristic of NPS therapy that is made possible by the ultrashort nature of this energy pulse. Because the lipid bilayers surrounding organelles are similar to the plasma membrane, their breakdown potential should also be 400–500 mV. However, the magnitude of the imposed electric field required to generate 500 mV across a very small organelle is much larger than that needed for a plasma membrane. For example, generating 500 mV across both membranes of a mitochondrion that is 0.5 μm wide requires a field of 1 V/0.5 µm which equals 20 kV/cm. The electric pulses typically used to permeabilize cells are 100 µs long and 1 kV/cm. Applying fields 20-fold larger than this would deliver so much energy that the target cell would exhibit hyperthermia. However, nanosecond pulses are 1000-fold shorter and so deliver 1/1000th of the energy for the same current and voltage. That is the main reason that it is possible to use NPS treatment to generate fields large enough to permeabilize organelles in a nonthermal manner.

This ability to permeabilize intracellular organelles is a very powerful way to influence cellular function. For example, consider the mitochondria that generate 200 mV across their inner membrane to drive ATP synthesis. When their membrane permeability is increased, this potential gradient and the consequent ATP synthesis are greatly reduced. Reducing the energy supply in cells can clearly have profound effects on all cell functions. In fact, this permeabilization of the mitochondria results in the entry of water and swelling that contributes to the initialization of the regulated cell death path. In summary, the mechanism by which NPS therapy affects the skin is completely different from the thermal necrosis induced by other ablation modalities. NPS therapy triggers the cells’ built-in regulated cell death pathway in the cellular components of skin. The epidermal cells slowly die to become a crust that falls off revealing a new layer of epidermal cells. Only the tissue within the applicator electrodes is exposed to the electric pulses so the response is highly localized and does not have any effect on acellular structures such as collagen.

## Materials and Methods

### Devices used

For cryoablation, the Brymill liquid nitrogen applicator with a 1 cm flat probe (Part No. 205-1) was used. It was primed and applied to the skin for 3 to 4 s. For Bovie^®^ RFA, the Bovie Derm 102 was used with a 3/16” ball electrode (Part No. ES20) and a return grounding pad (Part No. ESRE-1) on a Bovie setting of 10 with an endpoint determined by Dr. Kaufman. Nanosecond pulsed electric fields were applied with the CellFX™ system (Pulse Biosciences, Hayward, CA) primarily using a 5 × 5 mm microneedle applicator depositing 0.1 mJ/mm^3^. Before the CellFX treatments, 2% lidocaine injections were applied to facilitate patient comfort during energy delivery. Treatment tips measuring 5 × 5 mm, 7.5 × 7.5 mm, and 10 × 10 mm were used and were composed of two parallel rows of 2 mm-long microneedles spaced 5, 7.5, and 10 mm apart, respectively.

### Subjects

Subjects provided written informed consent, and the study conforms to the U.S. Federal Policy for the Protection of Human Subjects. Two subjects were screened and enrolled by the principal investigator from patients already scheduled to undergo elective abdominoplasty surgery. Subjects presented with healthy abdominal skin, a normal range of skin laxity, and no history of skin treatments on the abdomen. Biomedical Research Institute of America Institutional Review Board (IRB) reviewed the study protocol and provided oversight for this non-significant risk study. A total of 30 areas, 14 of spot sizes of 25 mm^2^, 12 of spot sizes of 56.25 mm^2^, and four with spot sizes of 100 mm^2^ within the planned excision area were treated at 2 h, 4 h, 1 day, 15 days, 30 days, and 90 days before surgery. Photographs of each treated area were taken over time, and biopsies of surgically removed tissue were processed and evaluated for tissue changes using hematoxylin and eosin, trichrome, Caspase-3, microphthalmia-associated transcription factor, and Elastin stains and evaluated by a dermatopathologist.

### Study design

The study was designed to meet two main objectives as follows: (1) document the clinical changes in healthy human skin following NPS exposure and exposure to the two gold standards of cryoablation and BOVIE RFA therapy, and (2) to perform a histological analysis of the treated tissue. Study objectives were achieved by treating healthy abdominal skin in a predefined grid pattern with NPS therapy, cryotherapy, and Bovie^®^ RFA and comparing the clinical and histological changes in the skin. Samples collected on the day of surgery represented 2-h, 4-h, 1-day, 15-days, 30-days, and 90-days post treatment with the three energy modalities.

### Histological sample preparation

The abdominal skin targeted for resection was excised, using the tattoo dots to recover the original treatment locations of the six grids. Biopsies of each treatment area produced 30 full-thickness dermal samples, with two additional control samples just outside the treatment grids for each of the study subjects. Individual samples were placed in collection jars with 10% neutral formalin for preservation and shipped to a histopathology laboratory, Pinkus Dermatopathology Laboratory, for staining and evaluation by a dermatopathologist.

## Results

### Clinical findings

**FIG. 1.**
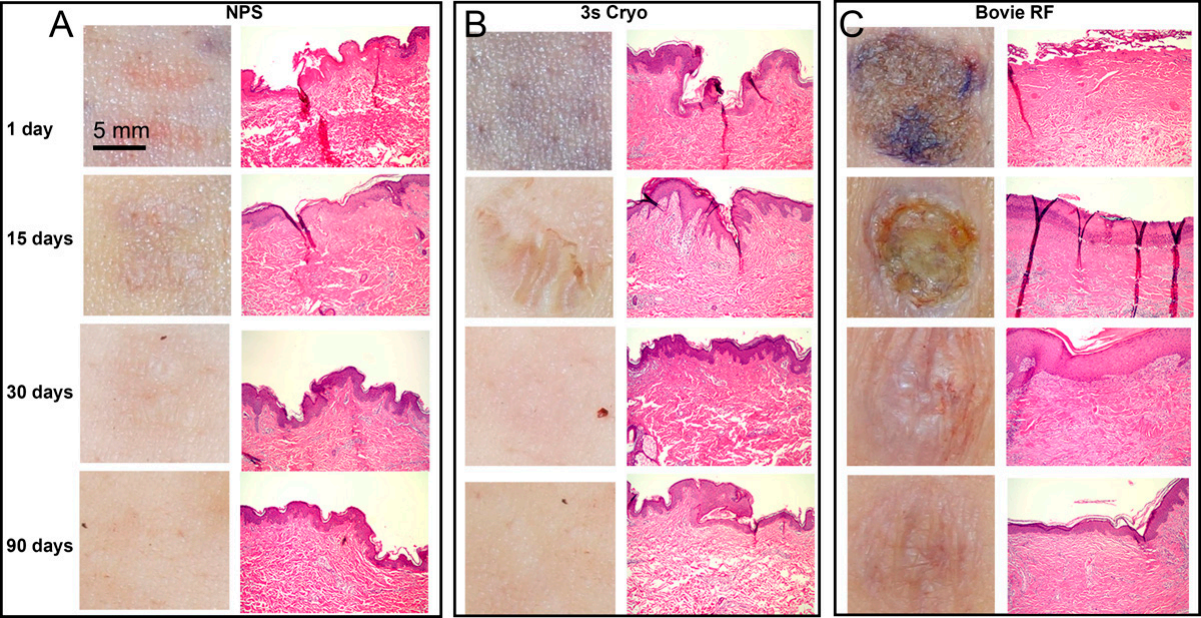
Surface photograph and corresponding histological section collected from subject 1’s abdominal skin treated with the method indicated at the top of each column at the time point shown on the left. **(A)** NPS therapy applied at 130 mJ/mm^3^ with a 5 × 5 mm tip; **(B)** Three seconds of cryoablation; **(C)** A few seconds of Bovie RFA ablation on a Bovie setting of 10. Scale bars on the upper left apply to all similar image types. RFA, radiofrequency ablation.

**FIG. 2.**
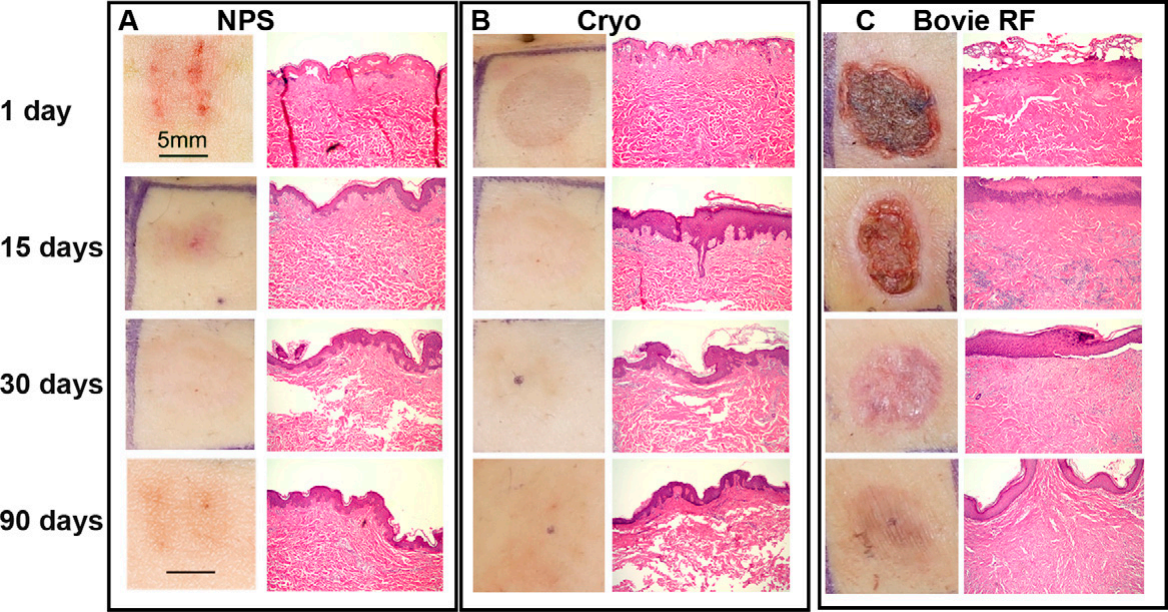
Surface photograph and corresponding histological section collected from subject 2’s abdominal skin treated with the method indicated at the top of each column at the time point shown on the left. **(A)** NPS therapy applied at 130 mJ/mm^3^ with a 5 × 5 mm tip; **(B)** Three seconds of cryoablation; **(C)** A few seconds of Bovie RFA ablation on a Bovie setting of 10. Scale bars on the upper left apply to all similar image types. RFA, radiofrequency ablation.

The appearance of the skin surface following each of the three treatments on day 1 was radically different. The most striking was the brown crust generated by the Bovie^®^ RFA device (Figs. 1 and 2). It was still present 15 days later and left a visible scar at both 30 and 90 days for the radiofrequency-treated tissue. Cryoablation generated a circular mark of hypopigmented and hyperpigmented skin which faded and appeared to exhibit mild hyperpigmentation at 90 days. “Track” marks resembling the microneedle tip array were apparent along with clear erythema on day 1 for NPS-treated tissue, which faded by 15 days with residual mild hyperpigmentation at 90 days.

### Histological findings

The histological characteristics of each treatment modality were compared and showed some distinctive differences. As expected, evidence of thermal damage was seen for RF-ablated and cryoablated tissue but there was no evidence of thermal damage for NPS-treated areas.

### Epidermis

**FIG. 3.**
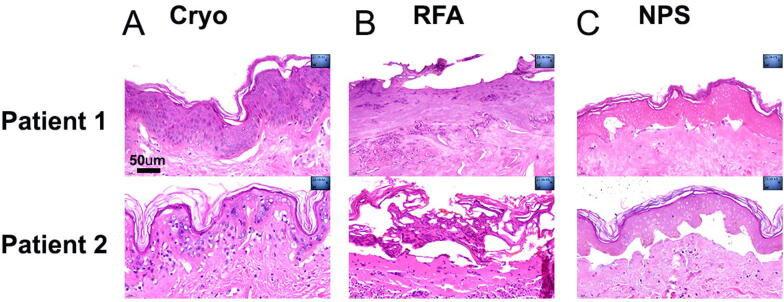
H&E-labeled thin sections of abdominal skin were collected 1 day after the indicated treatments. **(A)** Patient 1 and 2 skin treated with 3 s of cryo; **(B)** Patient 1 and 2 skin treated with a few seconds of Bovie RFA; **(C)** Patient 1 and 2 skin treated with NPS. The scale bar on the upper left image applies to all images. NPS, nano-pulse stimulation; RFA, radiofrequency ablation.

At 1 day post treatment, NPS-treated area exhibited characteristic ghost-like unstained nuclei and intact outer cell membrane (Fig. 3). In contrast, treatment with cryoablation showed dyskeratosis and epidermal necrosis at 1 day, and RF-treated epidermis showed acute necrosis with elongated nuclei. By 15 days post treatment, both NPS- and cryoablation-treated areas showed complete epidermal recovery, while the RF-treated areas showed on average 50% epidermal recovery at 15 days post treatment and full recovery by 30 days post treatment.

**FIG. 4.**
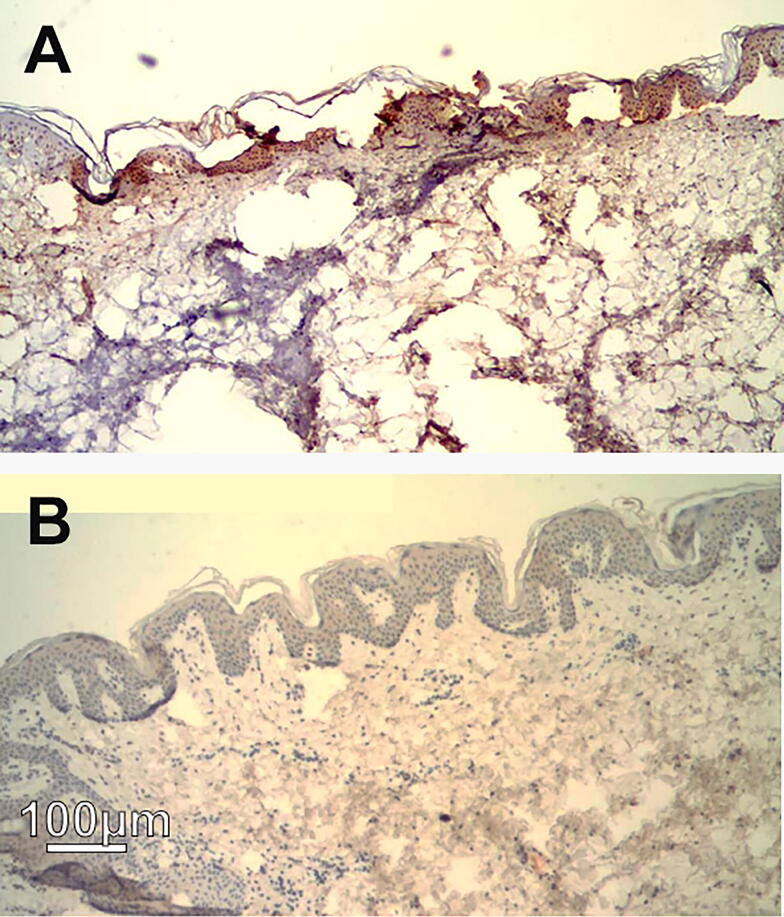
Immunohistochemical labeling with antiactivated Caspase-3 of thin section of abdominal skin collected 2 h post NPS treatment. **(A)** 107 mJ/mm^3^ NPS applied. The brown stain indicates the presence of activated Caspase-3. **(B)** 45 mJ/mm^3^ was applied to this sample which is insufficient to initiate regulated cell death and lacks activated Caspase-3. The magnification is indicated by the scale bar and applies to both **(A)** and **(B)**. NPS, nano-pulse stimulation.

Because the NPS-treated skin exhibited the ghost-like nuclei characteristic of regulated cell death, specialty staining with murine antibodies to activated Caspase-3 was performed at two hours and four hours post treatment. Extensive label on the NPS-treated epidermis and dermis using anti-Caspase-immunohistochemistry was found (Fig. 4). Activated Caspase-3 is an executioner caspase that hydrolyzes intracellular proteins during regulated cell death and is not normally found in cells undergoing necrosis.

### Dermis

**FIG. 5.**
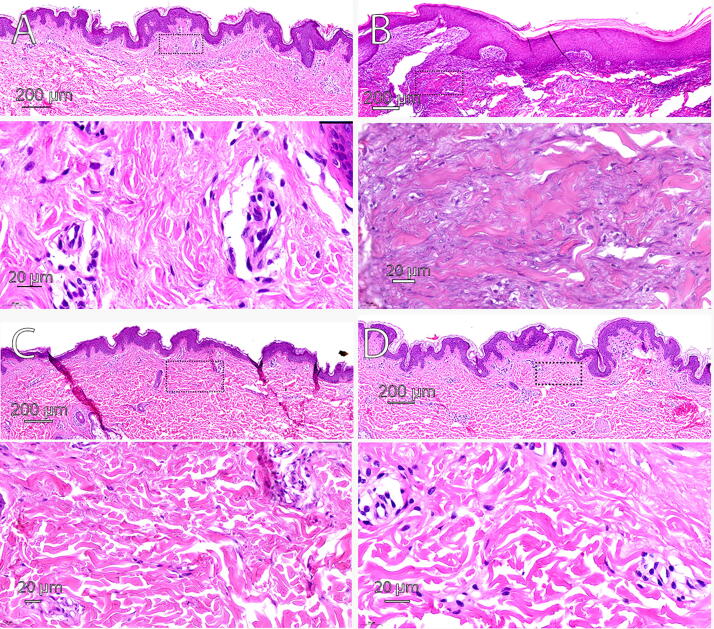
Thirty-day post treatment histology with H&E-labeled sections of abdominal skin from the same patient treated with the indicated energy along with an untreated control section for comparison. **(A)** Skin treated with 2 s of cryo with a magnified view of the dotted rectangle below; **(B)** Skin treated with Bovie RFA with a magnified image of the dotted rectangle below; **(C)** Skin treated with NPS with a magnified image of the dotted region below; **(D)** Untreated control sample for comparison with the others with a magnified dotted region below. NPS, nano-pulse stimulation; RFA, radiofrequency ablation.

In the NPS-treated skin, papillary and reticular fibrosis was absent, while cryoablated skin exhibited focal papillary dermal fibrosis and RF-treated skin showed deep dermal fibrosis with scar formation (Fig. 5). Elastic fibers were present and intact in both NPS- and cryoablation-treated skin but were reduced in RF-ablated skin. Within the deeper dermis, there was some loss of dermal adnexal structures initially in the NPS-treated skin followed by regeneration of small hair follicles and eccrine ducts by 90 days. The cryoablation-treated specimen showed changes limited to the epidermis and papillary dermis. In contrast, in RF-ablated skin, permanent loss of adnexal structures occurred down to the mid-reticular dermis to lower dermis. The average treatment depth measured 1 day post treatment for NPS-treated areas was 3.3 mm, while treatment depths for cryoablation samples were less than 1 mm. The Bovie^®^ RFA treatment depth extended to the mid-reticular dermis to the lower dermis, approximately 3 mm. Of note, the treatment depth measured for NPS-treated areas was based on the presence of adnexal structures, as NPS does not show a significant impact on the acellular dermis.

### Inflammation

**FIG. 6.**
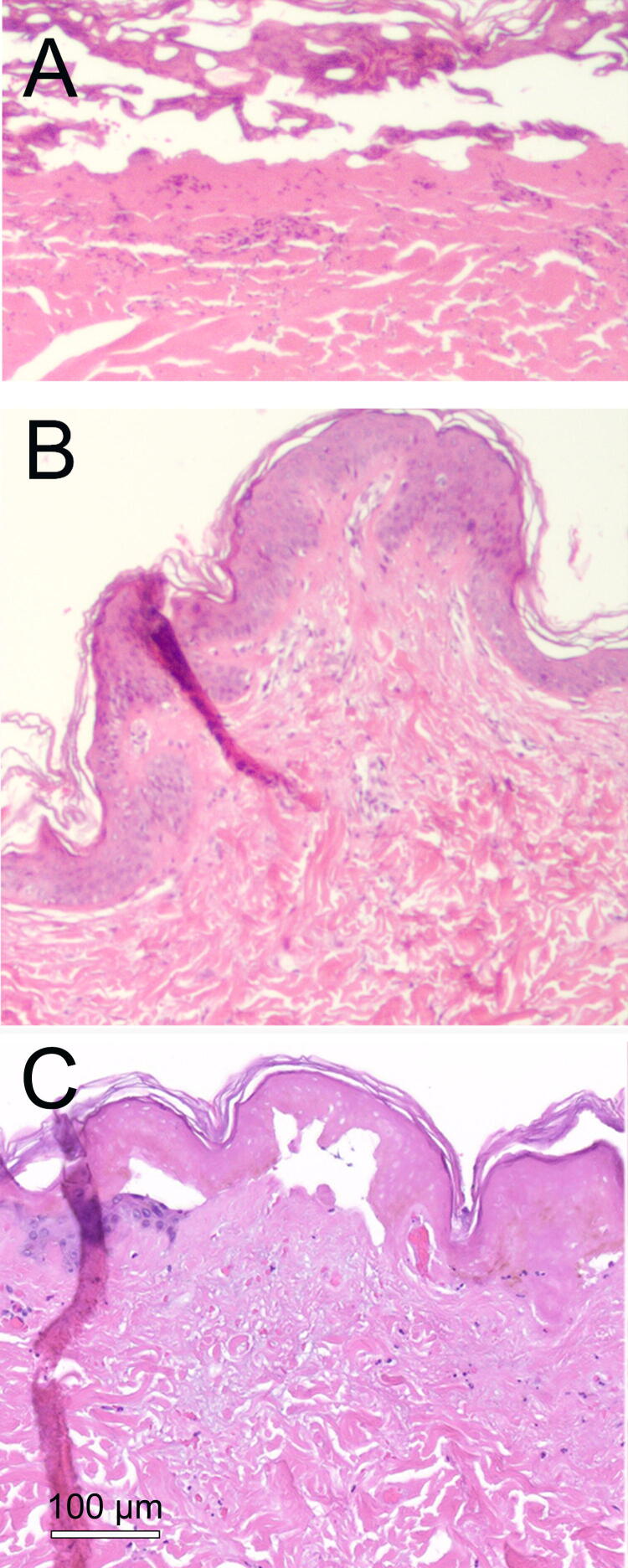
H&E-labeled thin sections of abdominal skin fixed 1 day after treatment. **(A)** Treated with a few minutes of RF; **(B)** Treated with 3 s of Cryo; **(C)** Treated with 107 mJ/mm^3^ of NPS. The blue specks in the dermis are lymphocytes and neutrophils that are much denser in the RF-treated skin than either the cryo- or NPS-treated samples. NPS, nano-pulse stimulation. In addition the lack of nuclear staining in the epidermis is indicative of regulated cell death or apoptosis.

Inflammation in the papillary dermis was minimal in the NPS- and cryoablation-treated skin in contrast to RF-treated skin which exhibited extensive inflammation in both the papillary and reticular dermis, showing resolution by 30 days post treatment (Fig. 6).

### Melanocytes

All three of these treatments resulted in a decreased melanocyte count by day 1, but in the NPS-treated skin, the melanocyte density returned to normal numbers by day 90, whereas only partial replacement of melanocytes occurred for the RF-ablated skin and only 20% of melanocyte count compared with controls for the cryoablated skin.

### Scar

Both subjects exhibited scar from the Bovie RFA on all the photo days post treatment, as well as histological evidence of fibroplasia and likely long-term scar in contrast to NPS treatments which did not exhibit any evidence of long-term scaring on any day post treatment (Fig. 5). One shortcoming of this study is that the cryo-contact probe was only placed on the skin for 3 s for consistency purposes, where in practice, application of cryo may vary significantly with uses of both contact and spray probes. The cryoablation method utilized may represent a shorter time than often used to ablate lesions.

**FIG. 7.**
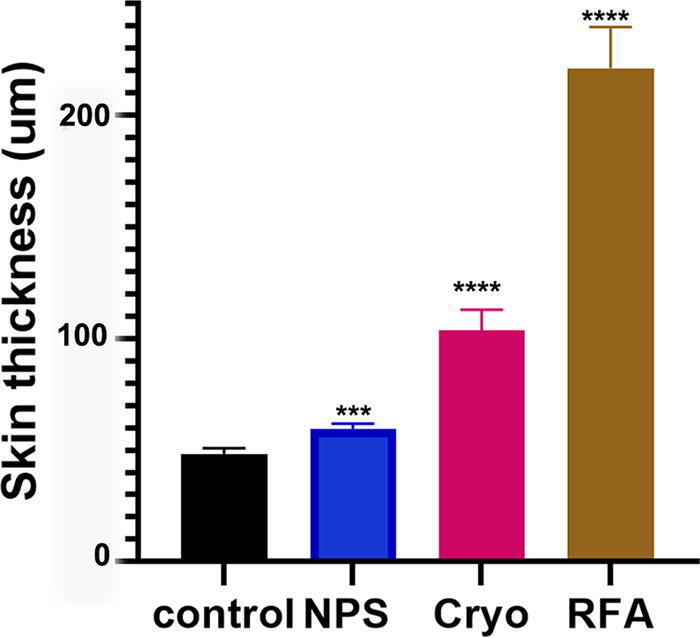
Mean thickness of skin 30 days after the ablation treatment. Significant difference from untreated control indicated: ***p < 0.003, ****p < 0.0001. Bar indicates SEM.

An analysis of the skin thickness 30 days after treatment shows that NPS-treated skin recovered to a mean thickness of only 25% thicker than the untreated skin, while cryoablated skin was twice as thick. RF-ablated skin was 3.5 times thicker than normal skin (Fig. 7). This further supports the conclusion that both RFA and cryoablation ablate skin by more damaging necrosis rather than regulated cell death.

## Discussion

Both the clinical and histological data illustrate the differences between the mechanism of cell death used by NPS therapy and that of either Bovie RFA ablation or cryoablation. These latter two utilize extreme temperatures to radically disrupt cellular membranes, as well as dermal fibers such as collagen, and initiate rapid necrosis. The disruption of collagen fibers can lead to scarring. In contrast, NPS treatments introduce very little heat and generate transient, 1-nm wide pores in the intact membranes to initiate the regulated cell death pathway that is built into all cells. NPS treatments have no effect on acellular components of the dermis such as collagen so usually do not result in scarring. The activation of this pathway results in a programmed cell death cascade in the epidermal cells that include the fragmentation of DNA, cleavage of proteins, nuclear pyknosis, and the translocation of “eat me” proteins such as calreticulin to the cell surface that attract dendritic cells.^6^ One day after the NPS treatment the nuclei of the epidermis have shrunk and do not take up stain in histological sections. Over the next week, these cells die and form a crust as new epidermal cells replace them. Several previous studies have documented this scarless regeneration of the epidermis within one week following NPS treatment.^17–21^

The NPS treatment modality shows high potential for treating cellular lesions that reside in the epidermis and dermis, with a low probability of scarring, and has already shown efficacy in treating sebaceous hyperplasia, seborrheic keratosis, warts, and other cellular-based lesions as referenced earlier. A range of microneedle depths of NPS treatment tips may be utilized to effectively target specific lesions. In contrast, the natural heat dispersion that accompanies both RF- and Cryo-ablation makes such localized treatments much more difficult.

### Limitations of these data

Only two subjects were treated in this study but the skin responses were similar in both of them so we feel that the conclusions resulting from these data are reliable.

In conclusion, the application of nanosecond pulsed electric fields to initiate regulated cell death in cellular structures is a new modality for the highly localized clearance of unwanted skin lesions with minimal scarring. It exhibits many advantages over the classical ablation modalities of burning and freezing the skin which often result in inflammation, fibrosis, and the permanent loss of adnexal structures.
